# Ball Milling and Magnetic Modification Boosted Methylene Blue Removal by Biochar Obtained from Water Hyacinth: Efficiency, Mechanism, and Application

**DOI:** 10.3390/molecules29215141

**Published:** 2024-10-30

**Authors:** Bei Wang, Yayun Ma, Pan Cao, Xinde Tang, Junliang Xin

**Affiliations:** 1 School of Chemical and Environmental Engineering, Hunan Institute of Technology, Hengyang 421000, China; abbywb@hnit.edu.cn (B.W.); 2021001043@hnit.edu.cn (P.C.); txd738011@126.com (X.T.); 2 School of Metallurgy and Environment, Central South University, Changsha 410083, China; 173501006@csu.edu.cn; 3 Dongjiang Environmental Protection Co., Ltd., Shenzhen 518104, China

**Keywords:** ball-milled magnetic biochar, dye, pyrolysis temperature, adsorption kinetics, DFT calculation

## Abstract

Ball milling is a feasible and promising method of biochar modification that can significantly increase its adsorption ability to methylene blue (MB). This study synthesized nine biochars derived from water hyacinth under different pyrolysis temperatures and modified with ball milling and Fe_3_O_4_. The structural properties of the pristine and ball-milled magnetic biochars were investigated and employed to adsorb MB. The results showed that ball milling significantly enhanced the specific surface area, total pore volume, and C-, N-, and O-containing groups of biochars, especially in low-temperature pyrolysis biochars. The Langmuir isotherm and the pseudo-secondary kinetic model fitted well with the MB adsorption process on biochars. After ball-milled magnetic modification, the adsorption capacity of biochar at 350 °C for MB was increased to 244.6 mg g^−1^ (8-fold increase), owing to an increase in accessible functional groups. MB removal efficiencies by low-temperature pyrolysis biochars were easily affected by pH, whereas high-temperature pyrolysis biochars could effectively remove MB in a wide pH range. WQM1, with the high adsorption capacity and stability, provided the potential to serve as an adsorbent for MB removal. Based on DFT calculations, the chemisorption and electrostatic interactions were the primary mechanism for enhancing MB removal with ball-milled magnetic biochar at low-temperature pyrolysis, followed by H-bonding, π–π interaction, hydrophobic interaction, and pore filling.

## 1. Introduction

With the growth of the printing and dyeing industry and the scarcity of clean water, wastewater treatment has become a research hotspot. China is one of the leading producers and exporters of dyes. Most raw materials, intermediates, and by-products in the dye industry are discharged as “three wastes”, accounting for approximately 10% of total industrial wastewater emissions [1]. Dye wastewater poses a significant threat to the biological environment and humans because to its high COD content, low biodegradability, and high chroma. Methylene blue (MB), as a typical azo dye, is widely used in textile dyeing, the manufacture of colored paper, medicines, and even animal husbandry [2]. MB and its metabolites will provide a health concern through carcinogenesis, teratogenesis, and mutagenesis [3,4]. Therefore, these industrial dye wastewaters must be treated before discharge.

Adsorption is considered as one of the most promising dye removal technologies in terms of efficiency, availability, and affordability [5]. Several adsorbents have been fabricated, including chitosan [6], metal oxides [7], SiO_2_ [8] and carbon-based compounds [9]. Using physicochemical modification to produce activated carbon from solid waste (e.g., sludge, biomass) is gaining popularity. The large specific surface area, porosity, and abundant functional groups aid activated carbon in the removal of pollutants from aqueous solutions [10,11]. In our earlier research, high-temperature pyrolysis combined with K_2_CO_3_ activation were used to create soybean-based activated carbon, and its maximum MB adsorption capacity was 434.78 mg g^−1^ [12]. To our knowledge, other studies have mostly concentrated on improving biochar’s adsorption capacity for pollutants, with little attention paid to preventing the contamination of the by-product and lowering the cost of physicochemical modification.

With a growing curiosity in ball milling technology, its combination with biochar materials opens up new opportunities for environmentally friendly and sustainable pollution control technologies [13,14]. When this high-energy mechanical movement in a ball mill reduces the size of the biochar to the micron or nanometer scale, it increases biochar’s surface area and uniformity [15]. Ball milling as a pretreatment can improve adsorption efficiency and shorten the equilibrium time of biochar and the time until metal and organic pollutant removal [16,17]. Researchers found that ball milling biochar can effectively remove two sulfonamide antibiotics (sulfamethoxazole and sulfadiazine) from wastewater, with the maximal adsorption capacity of 100.3 mg g^−1^ and 57.9 mg g^−1^, respectively [18]. A series of functional groups are produced by ball milling, which are beneficial to the adsorption of pollutants by biochar [19]. Furthermore, micro/nanoparticle biochar produced by ball milling possesses colloidal properties and high mobility, which increases Cd^2+^ transit in saturated porous media and adsorption [20]. Although ball milling biochar has many advantages as an adsorbent, the finer activated carbon powder is difficult to separate and recycle for later use.

To endow the biochar adsorbents with facile separation capabilities, a wide range of magnetic biochar has been created using chemical co-precipitation or pyrolysis activation with diverse iron sources, such as Fe, Fe_2_O_3_, and Fe_3_O_4_ [21]. The most common coprecipitation method has the advantages of a uniform particle size, good dispersion, and high loading capacity—while nonspecific precipitation will block the pores on the adsorbent and inactivated specific adsorption sites [22]. Although one-pot pyrolysis is simple, it requires larger energy and will lead to the uneven distribution of iron [23]. Ball milling technology has also been used to prepare magnetic biochar in recent years [24]. The magnetic ball-milled BC was obtained at 600 °C for MB adsorption capacity, and it showed a greater adsorption capacity than the usual magnetic activated carbon [25]. The pyrolysis temperature makes a difference to the physicochemical properties of biochar, since biochar obtained from industrial and agricultural wastes under high-temperature pyrolysis have better adsorption capabilities for pollution [26,27,28]. However, little research has been performed on how the one-step ball milling and magnetization affect the physicochemical properties and MB adsorption efficiency of biochar at different pyrolysis temperatures.

Water hyacinth (*Eichhornia crassipes*) is an aquatic weed that grows in rivers, lakes, and ponds all around the world. Its high resistance and reproductive abilities have caused great damage to water resources and the aquatic environment [29]. In terms of environmental preservation and resource use, this study uses the water hyacinth as a biomass material to create nine biochars by the pyrolysis and ball milling treatment for MB adsorption. The adsorption isotherms, kinetics, pH-dependence, and temperature affect were explored to assess their adsorption performance and adsorption process for MB. To further understand the adsorption mechanism, the pore structure and functional groups of the produced biochars were characterized by various techniques such as SEM-EDS, N_2_ adsorption/desorption isotherms, FTIR, XRD, XPS, and DFT calculation. Biochar’s regeneration and stability characteristics were investigated to determine its potential applications.

## 2. Results and Discussion

### 2.1. The Surface Structures of Different Biochars

**Figure 1:**
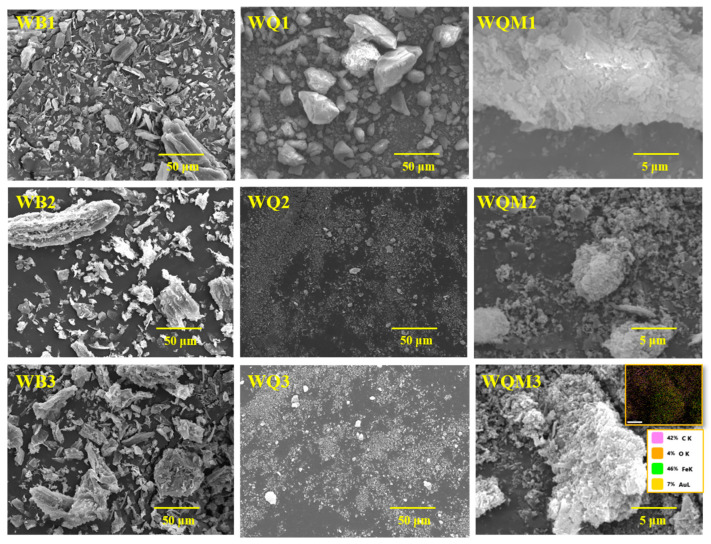
The detailed morphology of different biochars characterized by SEM.

The apparent morphology of biochars before and after ball milling at various pyrolysis temperatures is depicted in Figure 1. The unmodified biochars (WBx) demonstrate a distinct three-dimensional carbon skeleton structure. When the pyrolysis temperature increased, the biochar gradually loosened to develop apparent pores, mostly due to the ongoing degradation of organic components such as cellulose and hemicellulose in biomass at high pyrolysis temperatures [30]. Following ball milling, the biochar structure started to break and collapse, revealing a blocky and irregularly shaped fine structure. Ball milling reduced the particle size of WBx biochars from 50 μm to less than 1 μm, making it more evenly spread. The high-speed movement of balls in the ball mill mechanically decreased biochars particle size to the micron or nanometer scale, enhancing the surface area and homogeneity [15,31]. The EDS patterns show that the Fe element is successfully loaded and equally dispersed on the surface of WQMx biochar, confirming the feasibility of ball milled magnetic modification. This is also corroborated in the form of Fe_3_O_4_ by the XRD characterization of the ball-milled magnetic biochars (Figure S1).

**Figure 2:**
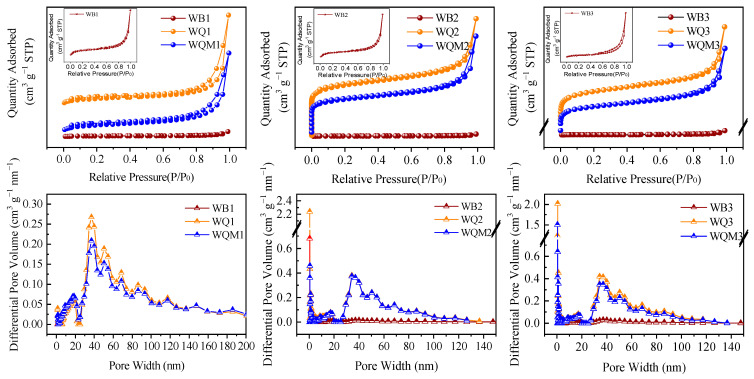
N_2_ adsorption–desorption curves and pore size distribution of biochars before and after ball milling.

The N_2_ adsorption–desorption curves of WBx, WQx, and WQMx biochars are displayed in Figure 2. According to the International Union of Pure and Applied Chemistry (IUPAC), the N_2_ adsorption–desorption curves belong to type Ⅳ with an H_4_ hysteresis loop, signifying the well-developed mesoporous structure [32]. WQx and WQMx biochars present the remarkable hysteresis loop, which prove a widely distributed mesopore. The pore size distribution also support that the presence of mesopore and macropore sizes between 20 and 100 nm in WQx and WQMx biochars. Furthermore, Table 1 shows that when the pyrolysis temperature increase, the specific surface area of WBx biochars does not vary much and remains less than 10. In contrast, WQx biochars have a significantly higher specific surface area and total pore volume, with mesopores accounting for the majority. These shapes allow macromolecules or ions to access the pores, creating ideal circumstances for the adsorption process. After ball-milled magnetic modification, the modest decrease in specific surface area and total pore volume of WQMx over WQx could be attributed to Fe_3_O_4_ doping into the pore structure of the biochars’ surface.

**Figure 3:**
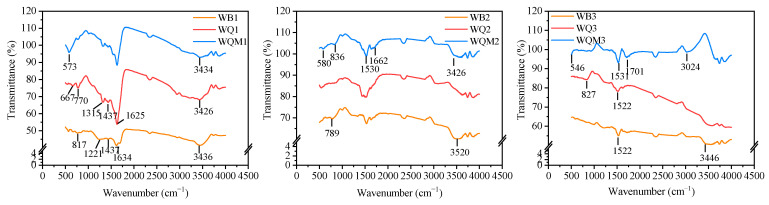
FTIR spectra of biochars before and after ball milling and magnetic modification.

Figure 3 demonstrates the FTIR spectra of biochars before and after ball milling and magnetic modification. As the pyrolysis temperature increases, the transmittance intensity decreases significantly, indicating the reduction in functional groups on the biochars surface. Various bands determine distinct functional groups. The visible bands in all biochars at 1625–1701 cm^−1^ are attributed to the C=O stretching in the aromatic ring, and the peaks in the range 770–836 cm^−1^ are assigned to the aromatic C-H stretching vibration of the aromatic compound [33]. The peak near 1530 cm^−1^ shows the asymmetric stretching vibration of N-H/N-O. The distinctive peaks at about 1221–1437 cm^−1^ in the low-temperature pyrolysis biochars correspond to stretching vibrations at C-H in saturated hydrocarbons or C-O in lipids. The visible bands exist in the range of 3024–3520 cm^−1^ and correlate to -OH, suggesting the presence of some hydroxyl groups on the surface of all biochars [34]. Furthermore, these peaks become more intense as functional groups are exposed during ball-milled magnetic modification, or the significant interactions with magnetic compounds. The vibration observed in the FTIR spectra of WQMx biochars between 546 and 580 cm^−1^ could be due to metal oxide (Fe-O) stretching [35]. A comparison of XPS profiles of WQx and WQMx biochars shows an increase in the percentage of oxygen (O1s) and iron (Fe2p) after the modification with Fe_3_O_4_ (Figure S2). The XPS spectra in WQ1 and WQM1, the C1s peak is deconvoluted into peaks at about 284.6 eV (C=C/C-C), 285.9 eV (C-O/C-N) and 289.1 eV (COOH), O1s peak is divided into -C=O (531 eV), -OH (532.5 eV) and -COOH (533.8 eV), the N1s peak includes pyridinic N (398.1 eV) and pyrrolic N (399.7 eV) [36]. In comparison, the high-temperature pyrolysis biochar WQ3 and WQM3 have enhanced graphitic N (401.1 eV) peaks and decreased C-O/C-N peaks and -C=O peaks in their high-resolution XPS spectra. The XPS spectra of Fe 2p is separated into four peaks at (Fe 2p) at 710, 711, 723, and 725 eV, corresponding to Fe^2+^ 2p_3/2_, Fe^3+^ 2p_3/2_, Fe^2+^ 2p_1/2_, and Fe^3+^ 2p_1/2_, respectively [37], proving that both Fe^2+^ and Fe^3+^ exist in the form of Fe_3_O_4_ in WQM1 and WQM3.

### 2.2. Adsorption Efficiency and Kinetics of MB by Biochars

The surface characteristics of biochars are influenced by the pyrolysis temperature, ball milling, and magnetic modification, which leads to varying MB absorption effectiveness. According to the results in Figure S3, the removal efficiencies of the biochars shows nonlinearly at the rise of pyrolysis temperature, with WB3 > WB1 > WB2. Furthermore, the high-temperature pyrolysis shortens the time it takes for the biochars to reach adsorption equilibrium. After the ball milling treatment, biochars present a considerable increase in MB removal, showing that WQ3 > WQ1 > WQ2. In contrast, following ball-milled magnetic modification, the removal efficiency of MB exhibit WQM1 > WQM3 > WQM2, which might be attributable to the fact that the magnetic modification produced the partial blockage of the pores and increased the number of functional groups on the biochar surface. The adsorption isotherm simulations demonstrate a better Langmuir model fit (Table 2), indicating that the biochars adsorption process is a monomolecular layer adsorption [38]. Overall, the low-temperature pyrolysis biochars after ball-milled magnetic modification retain a high saturation adsorption capacity (244.6 mg g^−1^), and the adsorption capacity of MB on WQM1 is comparable to that of magnetic biochars (Table 3).

**Figure 4:**
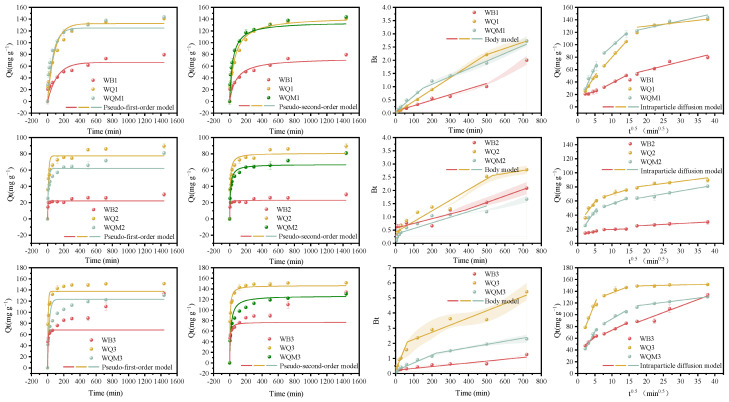
The pseudo-first-order, pseudo-second-order adsorption kinetic, Boyd models, and intraparticle diffusion model for the adsorption of MB onto various biochars.

Figure 4 and Table S1 depict various adsorption kinetic models and related parameters for MB adsorption by different biochars. The deviation between the simulated adsorption capacities of the pseudo-secondary kinetic and the experimental adsorption capacities is smaller and the R^2^ is greater. This indicates that the pseudo-second kinetic model is better suited to explaining the biochars’ adsorption process. The rate-limiting stage of MB adsorption on biochar is assumed to be chemisorption, which is the process of transferring, exchanging, or sharing electrons on the surface of biochar to establish a chemical bond [39]. The richer functional groups kept at low-temperature pyrolysis biochars play a crucial role. In addition, Boyd’s model is constantly applied to analyze which is the rate-limiting step. The Boyd plot is linear but does not pass through the origin, implying that the adsorption process is governed by liquid-film diffusion or chemical reaction [40]. According to the intraparticle diffusion model, the adsorption process of MB by the biochar was split into three stages: surface adsorption, progressive diffusion in the pores, and adsorption equilibrium [41]. It is worth noting that the adsorption rate of the WQx and WQMx biochars in the first stage is 1.4–6.6 times higher than that in the second stage. This may be due to the stronger adsorption driving force and more active adsorption sites supplied by the larger surface area after ball milling.

### 2.3. Effect of Temperature on MB Adsorption

The effect of varied reaction temperatures on the adsorption of MB by various biochars was studied. Figure S4 and Table S2 show that the MB adsorption capacities of WBx, WQx, and WQMx biochars increased from 25 °C to 55 °C, and the adsorption rate of biochar was faster under a high solution temperature at the beginning of adsorption. The first-order and second-order adsorption kinetics of MB on biochars at different temperatures show that the second-order kinetics still had a better fitting effect. However, the simulative adsorption rate (k_2_) of biochars fluctuated randomly with the increase in temperature. For example, the k_2_ of WQ1, WQM1, and WQ3 increase, but the k_2_ of other biochars may decrease with increasing temperature. Elevated temperatures often alter adsorption kinetics; however, the effect varies depending on the complexity of the adsorbent. Sometimes, high temperatures can raise the thermal kinetic energy of molecules, making them more accessible to adsorption sites and thereby accelerating the rate of sorption [42]. Different biochars have diverse pore shapes, chemical compositions, and surface functional groups, which each may have an impact on the adsorption efficiency.

### 2.4. Effect of pH on MB Adsorption

**Figure 5:**
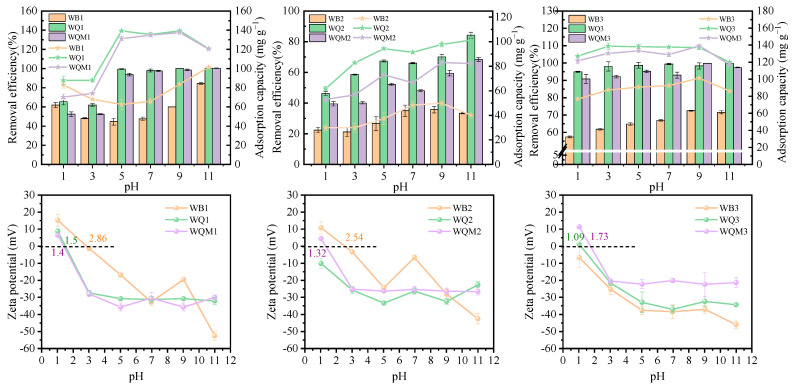
Effect of pH on the removal efficiency and adsorption capacity of MB by biochars, and the Zeta potential evolution with pH for biochars.

The solution pH can influence MB adsorption by regulating the surface charge and degree of ionization of the biochars. As the pH of the solution rose from 1 to 9, Figure 5 shows that the adsorption capacity and removal efficiency of MB by biochar improve. On the other hand, an excessively high pH alters the composition of the MB solution and reduces its initial concentration. Because of the number of functional groups on its surface, low-temperature pyrolysis biochars were likely the most affected by pH. When the pH rises over the zero-point charge (pH_zpc_), the amino, carboxyl, and hydroxyl functional groups on the surface of biochar particles deprotonate [43]. Their negative charge then creates an electrostatic attraction with the positive charge of MB, improving the adsorption performance. In addition, the pH_zpc_ of the WQx biochars are smaller, indicating that its surface charges were more negative, facilitating the adsorption of the cationic dye MB [5,44]. For example, the potentials of WB1 and WQ1 are 2.86 and 1.5, respectively, and the adsorption capacity of WQ1 is twice that of WB1. The pH_zpc_ of all WQMx biochars were increased, resulting in a modest decrease in the enhancing effect of the adsorption rate as compared to the WQx biochars. This outcome is in line with the previously given description. The electrostatic interaction between the dye and the surface of the biochar particles was a substantial factor in the pH_zpc_ of the biochar, which is below −27 mV overall.

### 2.5. Reusability of WQM1 and WQM3

The adsorption performance of ball-milled magnetic biochars (WQM1 and WQM3) for MB after five cycles is shown in Figure S5. The adsorption capacity of WQM1 and WQM3 for the MB dye gradually decreases with the increase in the number of cycles, from 50.50 mg g^−1^ and 50.48 mg g^−1^ to 47.27 mg g^−1^ and 41.72 mg g^−1^, respectively. And, the removal efficiency declines from 100% and 99.96% to 93.61% and 82.61%. The adsorption of MB dye by both ball-milled magnetic biochars still maintain good stability and adsorption capacity, with WQM1 outperforming. The reason may be that WQM1 mainly relies on chemisorption for MB due to abundant specific functional groups. While WQM3 adsorbs MB through pore filling, and part of the residual dyes in the pores during the elution process will affect its repeated adsorption performance. As a result, WQM1 performs well and has good stability when it is employed as a dye adsorbent.

### 2.6. Removal Mechanism Analysis

**Figure 6:**
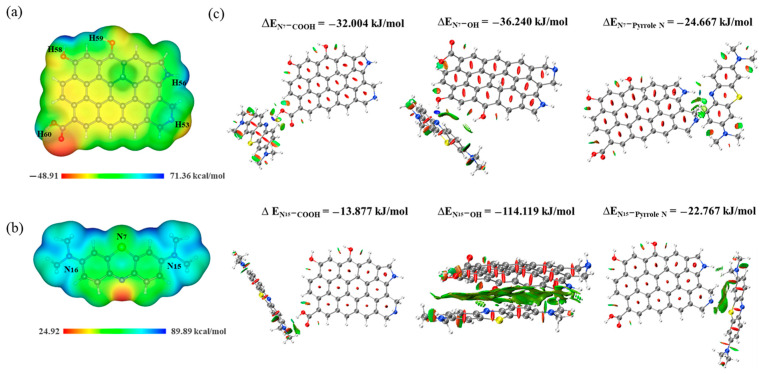
The electrostatic potential of WQ1 biochar (**a**) and MB (**b**), and the RDG and the binding energy of the interaction between WQ1 and the MB (**c**).

The electrostatic potential of MB and WQ1 are shown in Figure 6, and the scale from blue to red marks their upper and lower potential regions. The whole electrostatic potential of MB molecules presents a higher potential, indicating that it exhibits a positive charge in an aqueous solution and may be a target for nucleophilic assault [45]. By combining the electrostatic potential and natural population analysis atomic charge (Figure S6) of WQ1 biochar, it can be seen that the biochar’s negative charge region is mainly distributed near the functional groups of -COOH, -OH, pyridine N, and pyrrole N. And, these functional groups could be a target for nucleophilic assault [46]. Therefore, the biochar can adsorb MB in an electrophilic and nucleophilic manner and electrostatic interactions. WQ1 containing functional groups had a higher adsorption efficiency than other biochars, implying that the chemical bonding and electrostatic interaction are the key driver of MB adsorption by biochars. The results of adsorption kinetics and pH influence further verify the importance of these two effects.

**Figure 7:**
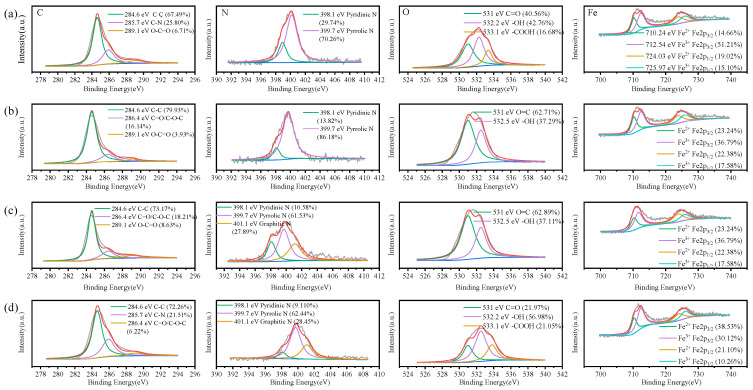
XPS spectra of WQM1 (**a**,**b**) and WQM3 (**c**,**d**) biochar before and after ball milling and magnetic modification.

**Figure 8:**
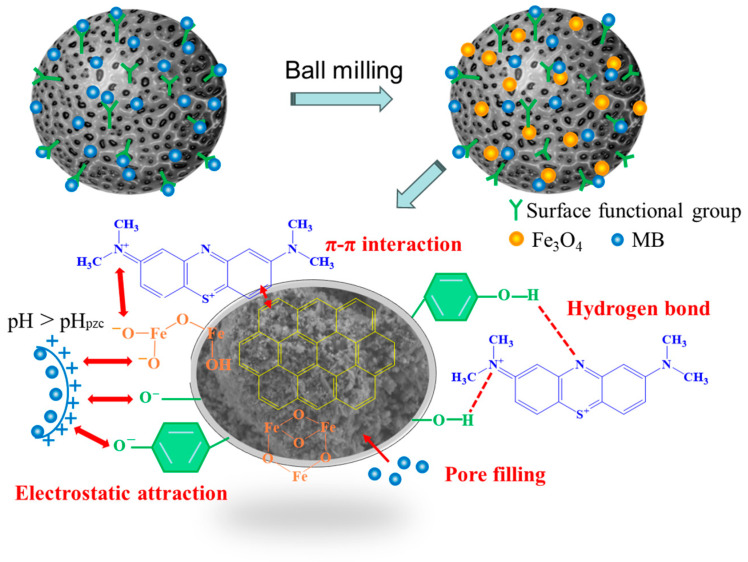
The proposed adsorption mechanisms of MB onto ball-milled biochar and ball-milled magnetic biochar.

The RDG analysis further discusses the types of weak interactions between WQ1 and the MB. If the color of the interaction region is blue, it is the strong attraction like a hydrogen bond, and green is the weak attraction such as π–π interaction and van der Waals force [47,48]. It can be seen from Figure 6c that the adsorption of MB by biochar is hydrogen bonding, which mainly depends on the interaction between -COOH, -OH functional groups in WQ1 structure, and C–N_7_ in MB. A reduction in these functional groups on WQM1 and WQM2 biochars following MB sorption in the XPS spectra also prove the interactions via hydrogen bonding (Figure 7) [49]. And the interaction between other functional groups is green, indicating that the adsorption between biochar and MB includes π–π interaction and van der Waals force. According to the model, the adsorption energy of MB molecules on the local functional groups of WQ1 adsorbents was estimated [50]. The quantum chemical calculation results indicate that the order of binding energy between WQ1 and MB is as follows: E_N15-OH_ < E_N7-OH_ < E_N7-COOH_ < E_N7-Pyrrole N_ < E_N15-Pyrrole N_ < E_N15-COOH_. The smaller the bonding energy, the stronger the adsorption force [46]. The result is consistent with the above RDG analysis, and the -OH functional group exhibits a high MB binding ability. Finally, the role of pore filling is significant and cannot be overlooked. Our analysis of the structure and adsorption performance of various biochars demonstrated that a specific surface area and mesopore volume are critical in MB removal. The adsorption mass transfer process of MB onto BK800 is influenced by the interfacial diffusion and intergranular diffusion, with interfacial diffusion being the primary adsorption process. In summary, it is hypothesized that the probable mechanism of MB adsorption by biochar include electrostatic interactions, hydrogen bonding, π–π interactions, hydrophobic interaction, and pore filling, as shown in Figure 8 [51,52].

The ball milling treatment is helpful for the exposing of the surface functional groups, which further enhanced the electrostatic adsorption ability of ball-milled biochar. The Fe_3_O_4_ modification enrich the oxygen-containing functional groups on the surface of the biochar, while the biochar’s strong magnetic characteristic facilitate recycling. As a result, the ball-milled magnetic biochar exhibit outstanding adsorption efficiency and reuse properties. In the future, the green synthesis of biochar nanoparticle adsorbent and the industrial scale application should be explored for drinking and wastewater treatment.

## 3. Experimental Section

### 3.1. Preparation of Ball-Milled Magnetic Biochars

Water hyacinth was collected from the lake near the school of Hunan Institute of Technology and rinsed with ultrapure water to remove surface impurities. We cut the water hyacinth into little pieces and dried it to a consistent weight at 80 °C in a vacuum oven before shattering it mechanically and sieving it to less than 60 mesh powder. The biomass was carbonized for 2 h at 350 °C, 550 °C, and 750 °C, and then heated at a rate of 5 °C min^−1^ in a N_2_-flowing tube furnace. The obtained biochars were named WBx (WB1, WB2, and WB3, respectively).

To obtain ball-milled biochars, the designated WQx (WQ1, WQ2, and WQ3), the biochars and agate balls (diameter = 5.60 mm, 120 g) were combined in agate bottles (80 mL) at a ratio of 100:1. The mixture was then processed at a specific speed in a planetary ball mill, with the direction of rotation changing every 0.5 h for a duration of 5–7 h. Ultimately, magnetite (Fe_3_O_4_) was added to the biochar mixture at a mass rate of 1:3 and ball-milled under the same conditions as previously described, resulting in the production of ball-milled magnetic biochars, which were designated as WQMx (WQM1, WQM2, and WQM3).

### 3.2. The Adsorption Efficiency and Kinetic Model

The additions of 0.1 g of biochar, magnetic biochar, and ball-milled biochar at varying pyrolysis temperatures were made to 150 mL of MB solution containing 100 mg/L. Conical flasks were put in a shaker set to shake at 140 rpm at 25 °C. Samples were taken at intervals of 0, 5, 10, 20, 30, 60, 120, 200, 300, 500, and 720 min. After filtering the sample using a 0.45 μm needle filter, the absorbance of the sample was measured at 679 nm using a UV spectrophotometer (UV-1800, AuCyBest, Shanghai, China). Finally, the removal rate (R, %) and the adsorption capacity (Qe, mg g^−1^) of the biochar material for MB were calculated by Equations (1) and (2).

(1) $$R=\frac{\left(C_{0}-C_{t}\right)\times 100\%}{C_{0}}$$

(2) $$Qe=\frac{V\times (C_{0}-C_{e})}{M}$$

where C_0_ is the initial concentration of MB solution (mg/L); C_t_ is the concentration of an MB solution at a specific time of adsorption, and C_e_ is the equilibrium concentration at which adsorption is complete (mg/L); V and M represent the volume of solution (L) and the mass of adsorbent (g), respectively.

To further investigate the adsorption mechanism, the adsorption characteristic constants were calculated using pseudo-first-order and pseudo-second-order kinetic models. And, the rate-limiting step in the adsorption process was determined by intra-particle diffusion model and Boyd model.

### 3.3. Adsorption Isotherm Model

The adsorption isotherms were evaluated using an MB concentration gradient (10, 20, 40, 60, 80, 100, 150, 200, 300, 500 mg/L). The adsorption conditions were the same as before, and Ce and Qe were determined after the adsorption was complete (24 h). In the early adsorption isotherm simulations, we found the two-parameter model (Langmuir, Freundlich, and Temkin isotherm model) is more representative than the three-parameter models (Toth and Langmuir–Freundlich isotherm model) [53]. So, the Langmuir and Freundlich models with a relatively good fit were chosen to represent the adsorption process and maximum adsorption capacity of biochar.

In addition, the adsorption process of MB by the material was simulated at different temperatures (25, 40, and 55 °C) and the thermodynamic parameters of the adsorption process were calculated.

### 3.4. Regeneration Studies

Following the adsorption of MB, the regeneration of WQM1 and WQM3 was assessed for five cycles. Moreover, 0.1 g of biochar was added to 50 mL of MB solution (100 mg/L) for each re-adsorption investigation, and the mixture was shaken for 24 h at 25 °C. A magnet was used to separate and collect the adsorbed biochar, which was then dried to a consistent weight. The adsorbent was further regenerated in hydrous ethanol by shaking for 2 h. After washing and drying, the adsorbent began its second adsorption cycle.

### 3.5. Characterization of Various Biochars

The elemental distribution and shape of various biochar materials were examined by scanning electron microscopy (SEM, JSM-6360 LV, JEOL, Tokyo, Japan) with energy-dispersive X-ray spectroscopy (EDS).

The adsorption and desorption isotherms of N_2_ were determined at −196 °C using an ASAP2020 fully automated specific surface and porosity analyzer (Micromeritics, Norcross, GA, USA). Using density functional theory (DFT) and the Brunauer–Emmett–Teller (BET) theoretical calculation, the specific surface area, pore volume, and pore size distribution of the biochar materials were also calculated.

Additionally, the distribution of functional groups on the surface of biochar was identified by Fourier transform infrared spectroscopy (FTIR-8700, Shimadzu, Tokyo, Japan) and X-ray photo-electron spectroscopy (XPS, Thermo Scientific K-Alpha, Waltham, MA, USA).

### 3.6. DFT Calculations

The WQ1 biochar and MB structural models were constructed, and all-electron DFT calculations were carried out by the latest version of ORCA quantum chemistry software (Version 5.0) [54]. For the geometric optimization of ground-state structures, the corrected version of r2SCAN exchange-correlation functional proposed by Grimme (so-called r2SCAN-3c) was adopted. The singlet point energy calculations were performed with the B3LYP functional and the def2-TZVP basis set. The interaction between the WQ1 and the MB was studied in the water using the RDG (reduced density gradient) through Multiwfn and VMD software (Version 3.8(dev), update date: 29 July 2024). The DFT-D3 dispersion correction with BJ-damping was applied to correct the weak interaction to improve the calculation accuracy. The adsorption energy (∆E) between WQ1 and the MB was calculated by the following formula [55].

∆E = E_com_ − (E_MB_ + E_WQ1_)

where E_com_ is the complex energy of the interaction between WQ1 and MB, E_MB_ is the energy of MB molecule, and E_WQ1_ is the energy of WQ1. The geometrical counterpoise correction was used to remove the artificial over-binding effects from the basis set superposition error. The SMD implicit solvation model was used to account for the solvation effect [56].

## 4. Conclusions

This paper states that ball-milling treatment is conducive to enhance the MB absorption for biochar due to its highly porous structure and more exposed functional groups. Even low-temperature pyrolysis biochar can show excellent adsorption performance, like at high-temperature pyrolysis, both WQ1 and WQ3 removed up to 99% of MB within 12 h. The adsorption capacity of an adsorbent for MB is primarily determined by electrostatic adsorption at pH > pH_zpc_ and the hydrogen bonding interaction between C-N functional groups in MB and the -OH and -COOH groups on the adsorbent surface. Furthermore, one-step ball milling combined with magnetic treatment boosted the biochar removal efficiency while also increasing its recyclability. WQM1 provides good reusability and stable adsorption performance in the removal of MB, making it a viable adsorbent from both economic and environmental standpoints. This study will serve as a scientific and technical reference for the further treatment of dyeing wastewater by ball milling refining low-cost biochar adsorbents.

## Figures and Tables

**Table 1:** Specific surface area and pore structure parameters of various biochars.

Samples	BET Surface Area (m^2^ g^−1^)	Pore Volume (cm^3^ g^−1^)			Average Pore Size (nm)	XPS Atomic (%)			
		Total Pore Volume	Micropore Volume	Mesopore Volume		C	N	O	Fe
WB1	0.30	\	\	\	\	82.45	3.71	13.84	\
WQ1	30.53	0.14	0.00	0.14	18.74	83.24	3.68	13.09	\
WQM1	32.59	0.13	0.00	0.13	15.46	69.85	2.92	25.41	1.82
WB2	5.14	0.01	0.00	0.01	9.38	\	\	\	\
WQ2	378.75	0.42	0.12	0.30	4.46	\	\	\	\
WQM2	276.78	0.36	0.08	0.28	5.20	\	\	\	\
WB3	8.87	0.02	0.00	0.02	9.97	83.14	3.7	13.16	\
WQ3	509.38	0.50	0.18	0.33	3.95	83.77	3.88	12.35	\
WQM3	389.48	0.42	0.13	0.29	4.30	71.63	2.74	23.72	1.91

**Table 2:** Fitting parameters of Langmuir and Freundlich models for different biochars.

Pyrolysis Temperatures (°C)	Qe, exp (mg g^−1^)	Langmuir Model ^a^			Freundlich Model ^b^		
		Q_m_ (mg g^−1^)	K_L_ (L mg^−1^)	R^2^	K_F_	1/n	R^2^
WB1	29.81	31.54	5	0.92	13.28	0.19	0.64
WQ1	204.44	143.97	3.45	0.95	51.74	0.27	0.61
WQM1	244.58	197.59	0.62	0.94	107.23	0.13	0.67
WB2	13.58	14.13	0.08	0.97	4.72	0.21	0.80
WQ2	99.06	93.88	3.77	0.91	54.20	0.13	0.78
WQM2	93.18	80.69	3.29	0.95	58.91	0.11	0.62
WB3	76.98	29.91	18.28	0.91	18.30	0.22	0.96
WQ3	192.17	185.04	17.25	0.92	109.41	0.11	0.76
WQM3	177.6	137.49	27.94	0.79	94.08	0.10	0.64

a. $Qe=\frac{Q_{m}K_{L}C_{e}}{1+K_{L}C_{e}}.$; b. $Qe=K_{F}C_{e}^{\frac{1}{n}}.$ Q_m_ (mg g^−1^) is the maximum adsorption capacity of the sorbent, K_L_ (L mg^−1^) is the Langmuir constants related to the free energy of the sorption, and K_F_ (mg g^−1^) (L mg^−1^) and n are the Freundlich adsorption constant and a measure of adsorption intensity, respectively.

**Table 3:** Comparison of the adsorption capacity of MB onto various biochars.

Materials	Pyrolysis Temperature	Surface Area (m^2^ g^−1^)	Adsorption Capacity (mg g^−1^) for MB	Reference
Sludge-based magnetic biochar	600 °C	70.6→20.2	47.4	[21]
Ball-milled magnetic activated carbon	600 °C	544.9→75.4	304.2	[25]
Ball-milled magnetic hickory wood-based biochar	600 °C	319.1→90.6	500.5	[25]
Ball-milled magnetic straw-based biochar	450 °C	174.1→139.1	153.5	[13]
WQM1	350 °C	0.3→32.5	244.6	This study
WQM3	750 °C	3.9→389.5	177.6	This study

## Data Availability

Data are contained within the article and Supplementary Materials.

## Supplementary Materials

The following supporting information can be downloaded at: https://www.mdpi.com/article/10.3390/molecules29215141/s1, Table S1. Kinetic models’ parameters for the adsorption of MB onto various biochars. Table S2. The pseudo-first-order, pseudo-second-order kinetic models’ parameters for the adsorption of MB onto various biochars at different temperature. Figure S1. XRD distribution of three kinds of ball milled magnetic biochars. Figure S2. XPS distribution of biochars under low and high temperature pyrolysis. Figure S3. Removal efficiency, Langmuir and Freundlich adsorption isotherms of MB by different biochars. Figure S4. The pseudo-first-order, pseudo-second-order adsorption kinetic fitting of MB adsorption by biochars at different reaction temperature. Figure S5. The adsorption performance of MB onto WQM1 and WQM3 after five cycles. Figure S6. NPA (Natural population analysis) atomic charge of WQ1 biochar (a) and MB (b).
