# Supplementary material for: Ball Milling and Magnetic Modification Boosted Methylene Blue Removal by Biochar Obtained from Water Hyacinth: Efficiency, Mechanism, and Application
Source: Molecules. 2024 Oct 30;29(21):5141. doi: 10.3390/molecules29215141 (PMC11547763; doi:10.3390/molecules29215141)
Supplement: Supplementary file 1 [file molecules-29-05141-s001.zip › molecules-3281484-supplementary.pdf]

Supporting Information:

Ball Milling and Magnetic Modification Boosted Methylene Blue Removal by Biochar Obtained from Water Hyacinth: Efficiency, Mechanism, and Application

Bei Wang [1], Yayun Ma [2,3], Pan Cao [1], Xinde Tang [1] and Junliang Xin [1,*,†]

[1] School of Chemical and Environmental Engineering, Hunan Institute of Technology, Hengyang 421000, China; abbywb@hnit.edu.cn (B.W.); 2021001043@hnit.edu.cn (P.C.); txd738011@126.com (X.T.)
[2] School of Metallurgy and Environment, Central South University, Changsha 410083, China; 173501006@csu.edu.cn
[3] Dongjiang Environmental Protection Co., Ltd., Shenzhen 518104, China
* Correspondence: hsingjl@126.com
† Current address: Henghua Road 18, Zhuhui District, Hengyang 421002, China

Table S1. Kinetic models' parameters for the adsorption of MB onto various biochars

		WB1	WQ1	WQM1	WB2	WQ2	WQM2	WB3	WQ3	WQM3
	$Q_{e,\ exp}$ (mg g^{-1})	29.81	204.44	244.58	13.58	99.06	93.18	76.98	192.17	177.6
Pseudo-first-order[a]	k_1 (g mg^{-1} min^{-1})	0.008	0.012	0.015	0.212	0.088	0.083	0.167	0.149	0.048
	$Q_{e,\ cal}$ (mg g^{-1})	66.37	132.46	124.93	22.03	77.57	62.08	68.27	137.56	123.44
	R^2	0.83	0.92	0.89	0.89	0.96	0.89	0.97	0.99	0.94
Pseudo-second-order[b]	k_2 (g mg^{-1} min^{-1})	0.00011	0.00016	0.0002	0.015	0.0017	0.0015	0.0027	0.0014	0.0005
	$Q_{e,\ cal}$ (mg g^{-1})	73.96	144.09	135.42	22.98	80.8	67	76.95	146.39	126.89
	R^2	0.90	0.97	0.97	0.92	0.99	0.95	0.98	0.99	0.98
Intraparticle diffusion model[c]	k_{p1} (mg·g^{-1} $min^{0.5}$)	1.63	8.75	12.68	0.87	6.5	7.11	4.72	14.62	10.76
	k_{p2} (mg·g^{-1} $min^{0.5}$)	2.9	6.08	4.96	0.16	1.48	1.76	2.78	2.2	3.41
	k_{p3} (mg·g^{-1} $min^{0.5}$)	1.37	0.63	1.2	0.26	0.62	0.83	2.19	0.14	0.73
	C_1 (mg g^{-1})	16.68	6.19	0.47	12.46	26.29	10.31	38.15	46.36	17.58
	C_2 (mg g^{-1})	9.37	18.96	48.23	18.18	54.75	38.24	45.86	115.3	58.84
	C_3 (mg g^{-1})	31.43	117.33	102.47	20.31	69.35	49.35	48.93	146.69	102.66
	$(R_1)^2$	0.88	0.98	0.99	0.97	0.83	0.93	0.96	0.97	0.99
	$(R_2)^2$	0.99	1	1	0.93	0.99	0.99	1	1	0.97
	$(R_3)^2$	0.88	0.88	0.92	0.99	0.76	0.99	0.97	0.81	0.99

a. $Q_t = Q_e(1 - e^{-k_1 t})$ b. $Q_t = \frac{Q_e^2 * k_2 * t}{1 + k_2 * t * Q_e}$ c. $Qt = K_p t^{\frac{1}{2}} + C$

Body model: $Ft = \frac{Q_t}{Q_e}$, $Ft \geq 0.85$ $B_t = -0.4977 - \ln\left(1 - \frac{Q_t}{Q_e}\right)$

$Ft \leq 0.85$ $B_t = (\sqrt{\pi} - \left(\pi - \frac{Ft * \pi^2}{3}\right))^2$

Where k_1 and k_2 are the pseudo-first order and pseudo-second order kinetic adsorption rate constants, respectively, and Qt is the adsorbed capacity per unit mass of adsorbent at time t. Kp and C are the diffusion rate constants and boundary layer thickness, respectively.

Table S2. The pseudo-first-order, pseudo-second-order kinetic models' parameters for the adsorption of MB onto various biochars at different temperature

			Pseudo-first-order			Pseudo-second-order		
		$Q_{e, exp}$ (mg g^{-1})	k_1 (g mg^{-1} min^{-1})	$Q_{e, cal}$ (mg g^{-1})	R^2	k_2 (g mg^{-1} min^{-1})	$Q_{e, cal}$ (mg g^{-1})	R^2
WB1	25°C	67.67	0.019	53.70	0.82	4.31E-4	59.79	0.88
	40°C	82.11	0.033	63.81	0.82	3.76E-4	112.18	0.88
	55°C	125.51	0.031	102.30	0.78	5.42E-4	71.57	0.90
WQ1	25°C	124.13	0.014	113.97	0.93	1.43E-4	128.61	0.97
	40°C	134.88	0.027	122.50	0.95	2.65E-4	134.82	0.99
	55°C	139.67	0.055	125.84	0.94	5.62E-4	135.42	0.88
WQM1	25°C	122.03	0.028	111.49	0.95	3.48E-4	124.29	0.98
	40°C	126.95	0.032	113.62	0.94	8.87E-4	148.46	0.91
	55°C	153.16	0.084	140.31	0.93	3.06E-4	122.41	0.99
WB2	25°C	34.19	0.130	27.59	0.84	6.00E-3	29.48	0.91
	40°C	45.54	0.063	34.05	0.74	2.00E-3	37.07	0.92
	55°C	71.27	0.036	57.67	0.87	6.58E-4	64.29	0.91
WQ2	25°C	80.09	0.091	69.67	0.91	1.80E-3	74.05	0.91
	40°C	90.29	0.095	82.71	0.95	1.70E-3	87.59	0.99
	55°C	117.17	0.091	100.37	0.90	1.30E-3	106.85	0.96
WQM2	25°C	73.78	0.058	59.33	0.88	1.30E-3	63.26	0.95
	40°C	84.24	0.128	66.39	0.86	2.70E-3	70.14	0.92
	55°C	113.35	0.039	95.58	0.86	5.12E-4	103.12	0.93
WB3	25°C	77.70	0.050	66.70	0.88	1.00E-3	71.50	0.95
	40°C	100.75	0.094	76.55	0.80	1.60E-3	82.16	0.89
	55°C	117.39	0.090	92.68	0.76	1.30E-3	99.53	0.86
WQ3	25°C	140.90	0.093	138.82	0.99	1.10E-3	145.51	0.99
	40°C	144.77	0.104	140.00	0.98	1.20E-3	146.69	0.99
	55°C	144.68	0.120	135.31	0.94	1.40E-3	142.47	0.99
WQM3	25°C	135.07	0.098	119.34	0.84	1.20E-3	127.13	0.93
	40°C	138.68	0.041	126.75	0.95	4.31E-4	136.48	0.99
	55°C	142.75	0.037	135.50	0.98	3.43E-4	146.83	0.99

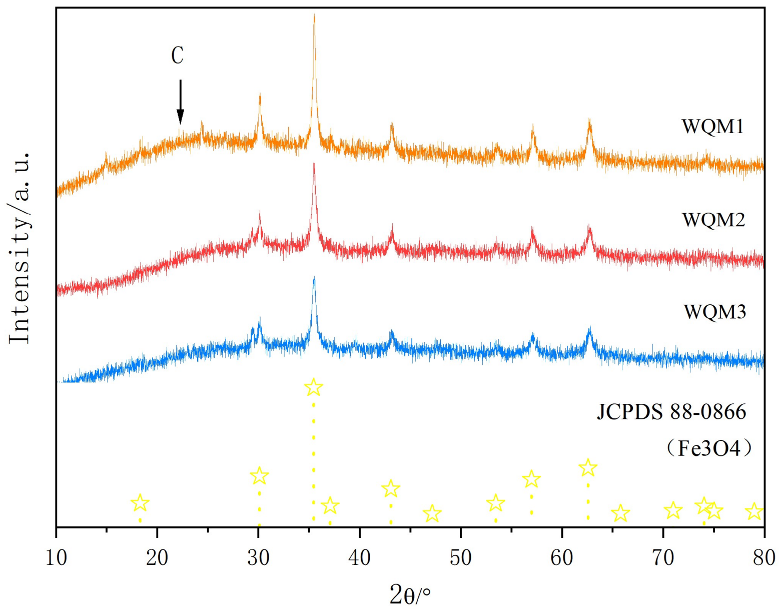

Figure S1. XRD distribution of three kinds of ball milled magnetic biochars

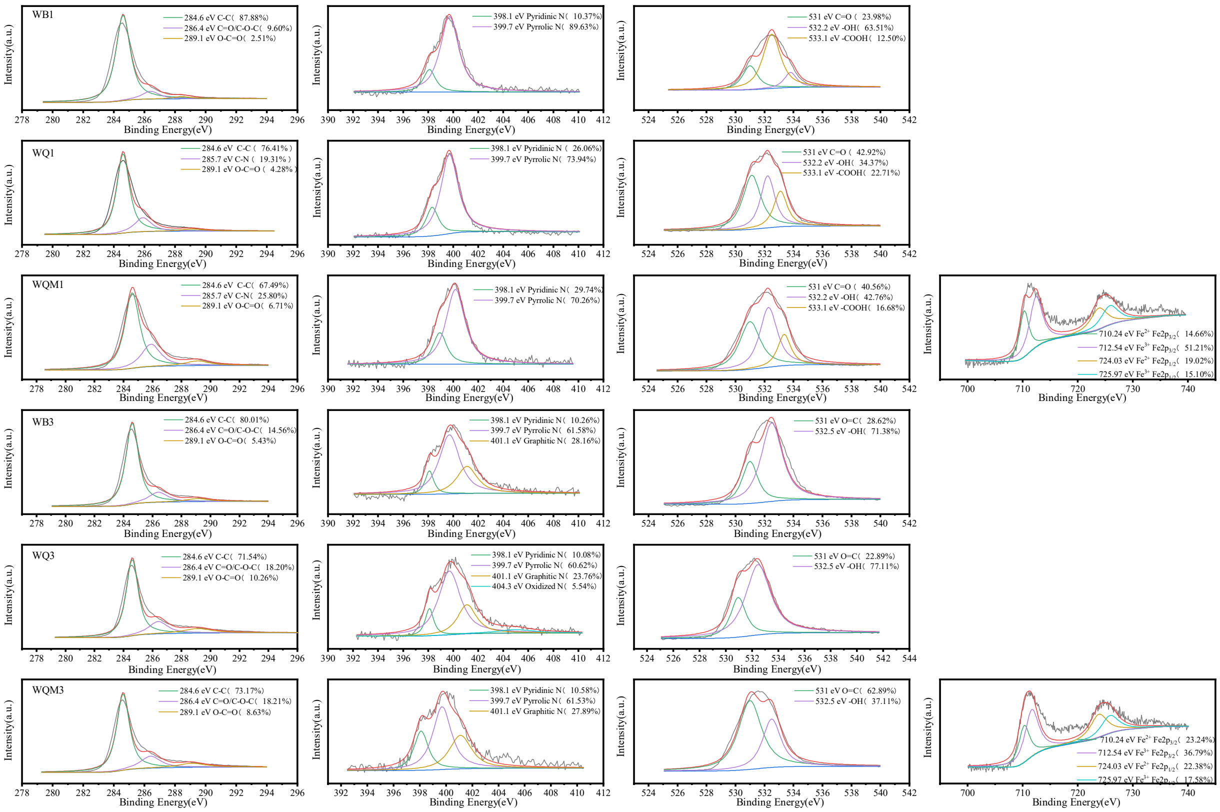

Figure S2. XPS distribution of biochars under low and high temperature pyrolysis

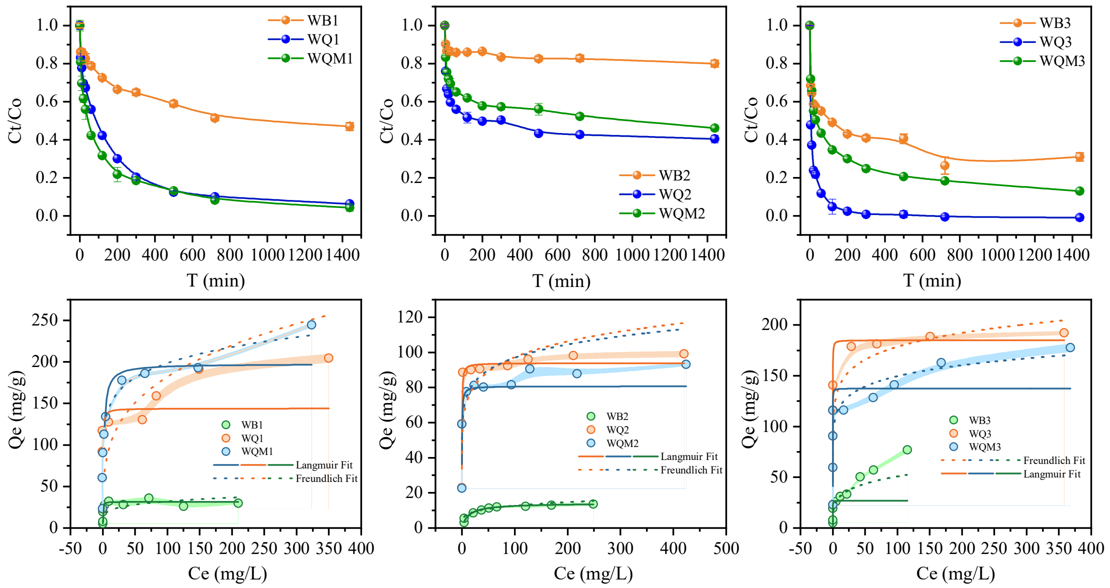

Figure S3. Removal efficiency, Langmuir and Freundlich adsorption isotherms of MB by different biochars

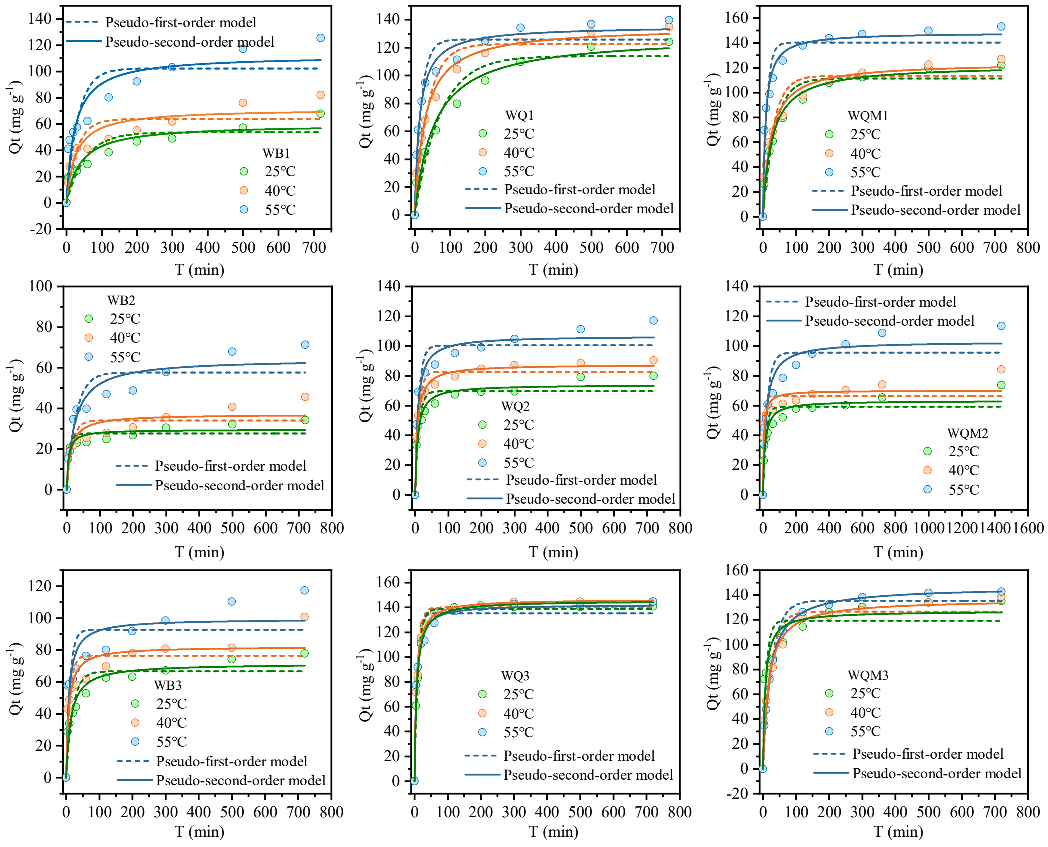

Figure S4. The pseudo-first-order, pseudo-second-order adsorption kinetic fitting of MB adsorption by biochars at different reaction temperature

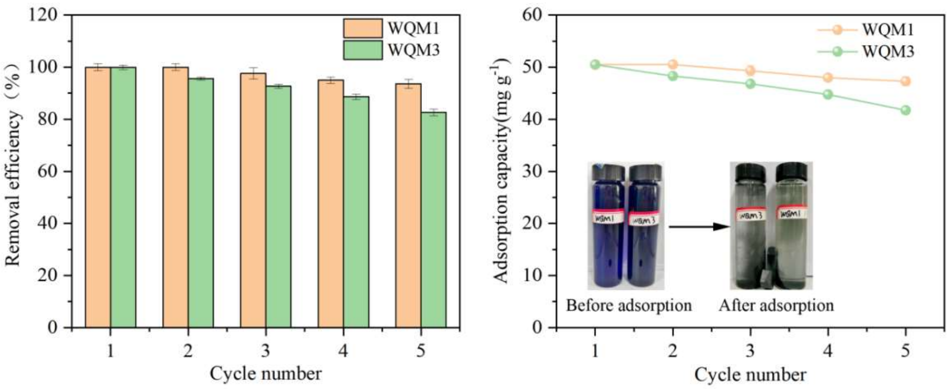

Figure S5. The adsorption performance of MB onto WQM1 and WQM3 after five cycles

(a)

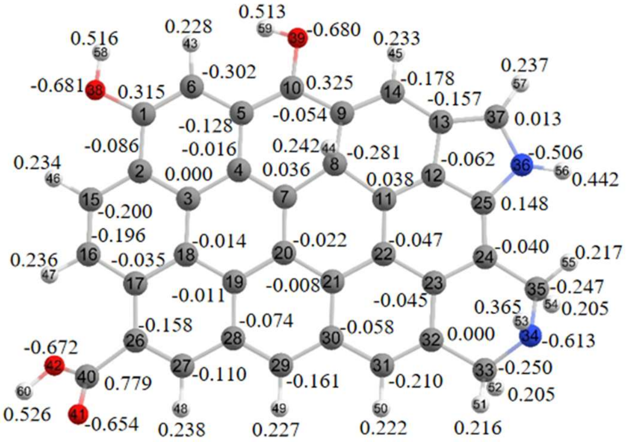

(b)

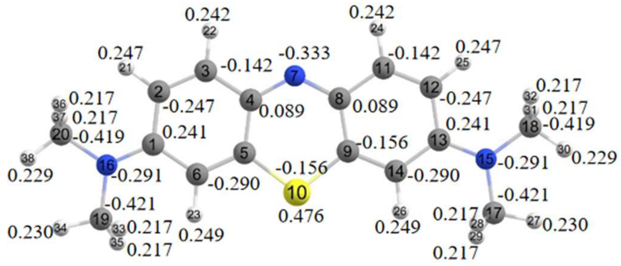

Figure S6. NPA (Natural population analysis) atomic charge of WQ1 biochar (a) and MB (b)
